# Effect of Diphtheria Toxin-Based Gene Therapy for Hepatocellular Carcinoma

**DOI:** 10.3390/cancers12020472

**Published:** 2020-02-18

**Authors:** Kenya Kamimura, Takeshi Yokoo, Hiroyuki Abe, Norihiro Sakai, Takuro Nagoya, Yuji Kobayashi, Masato Ohtsuka, Hiromi Miura, Akira Sakamaki, Hiroteru Kamimura, Norio Miyamura, Hiroshi Nishina, Shuji Terai

**Affiliations:** 1 Division of Gastroenterology and Hepatology, Graduate School of Medical and Dental Sciences, Niigata University, Niigata, Niigata 951-8510, Japan; t-yokoo@med.niigata-u.ac.jp (T.Y.); hiroyukiabe@med.niigata-u.ac.jp (H.A.); nsakai@med.niigata-u.ac.jp (N.S.); nagoya-takuro@med.niigata-u.ac.jp (T.N.); yujix4@gmail.com (Y.K.); saka-a@med.niigata-u.ac.jp (A.S.); hiroteruk@med.niigata-u.ac.jp (H.K.); terais@med.niigata-u.ac.jp (S.T.); 2 Department of Molecular Life Science, Division of Basic Medical Science and Molecular Medicine, School of Medicine, Tokai University, Isehara, Kanagawa 259-1193, Japan; masato@is.icc.u-tokai.ac.jp (M.O.); rascal511520531@yahoo.co.jp (H.M.); 3 Department of Developmental and Regenerative Biology, Medical Research Institute, Tokyo Medical and Dental University, Bunkyo-ku, Tokyo 113-8510, Japan; nm3081@cumc.columbia.edu (N.M.); nishina.dbio@mri.tmd.ac.jp (H.N.)

**Keywords:** hepatocellular carcinoma, gene therapy, diphtheria toxin fragment A, hydrodynamic gene delivery, alpha-fetoprotein

## Abstract

Hepatocellular carcinoma (HCC) is a major global malignancy, responsible for >90% of primary liver cancers. Currently available therapeutic options have poor performances due to the highly heterogeneous nature of the tumor cells; recurrence is highly probable, and some patients develop resistances to the therapies. Accordingly, the development of a novel therapy is essential. We assessed gene therapy for HCC using a diphtheria toxin fragment A (DTA) gene-expressing plasmid, utilizing a non-viral hydrodynamics-based procedure. The antitumor effect of DTA expression in HCC cell lines (and alpha-fetoprotein (AFP) promoter selectivity) is assessed in vitro by examining HCC cell growth. Moreover, the effect and safety of the AFP promoter-selective DTA expression was examined in vivo using an HCC mice model established by the hydrodynamic gene delivery of the yes-associated protein (YAP)-expressing plasmid. The protein synthesis in DTA transfected cells is inhibited by the disappearance of tdTomato and GFP expression co-transfected upon the delivery of the DTA plasmid; the HCC cell growth is inhibited by the expression of DTA in HCC cells in an AFP promoter-selective manner. A significant inhibition of HCC occurrence and the suppression of the tumor marker of AFP and des-gamma-carboxy prothrombin can be seen in mice groups treated with hydrodynamic gene delivery of DTA, both 0 and 2 months after the YAP gene delivery. These results suggest that DTA gene therapy is effective for HCC.

## 1. Introduction

Liver cancer is responsible for a great number of cancer-related deaths worldwide [1]. For hepatocellular carcinoma (HCC), which is responsible for >90% of primary liver cancers [2], various conventional therapeutic options are available, including surgical resection, ablation, chemoembolization, systemic chemotherapy, molecularly targeted agents, and liver transplantation [3,4,5,6]. However, since the consideration of the remaining hepatic function is essential in determining therapeutic options, said therapies are insufficient for advanced-stage liver cancer in terms of efficacy. To be sure, recent developments in the field of molecular targeted agent have shed light on chemotherapy for HCC [6] with respect to molecular expression differences in the tumor [7] and the potential promise for immune checkpoint inhibitors [8]; however, current therapies require further modifications to be fully effective [9,10,11]. This is partly due to the heterogeneity of tumor cells in HCC [12,13,14,15], which can cause a high risk of recurrence and drug resistance. Therefore, novel therapies are required for the effective treatment of advanced-staged HCC with poor hepatic reserve functions.

Recently, cancer gene therapy has developed in parallel with the significant improvement of genomic information using next-generation sequencing (NGS) and advances in the techniques of molecular biology [16,17] that use two-dimensional culture systems and patient-derived primary cancer cells [18,19] to target and modify tumor-related genes [20,21,22,23]. Therefore, innovative basic research and clinical trials focusing on the development of gene therapy for HCC are becoming more common [23,24,25,26]. The following cancer gene therapies for HCC have been tested in both basic and clinical research: the modification of genes related to tumor suppressors, oncogenes, suicide genes, those encoding the proteins expressed on the tumor cell surface, and the T-cell receptor to target the tumor, as well as genetic immunotherapy [17,24,25,27]. Among these strategies, we focus on the suicide gene delivery strategy, which was recently reported to be elicited in a tumor-specific manner using transcriptionally targeted retroviral replicating vectors [28], targeting the genomic rearrangement in the tumor by using the genome-editing approach to insert the suicide gene [29]. Suicide gene therapeutic strategies include the following: a combination of transgenes delivery that converts and administers prodrugs, such as herpes simplex virus thymidine kinase and ganciclovir [30]; delivery of cytotoxic genes; delivery of oncogene silencers, such as siRNA; delivery of genes expressing antibodies that block the tumor cell’s vital pathways, specifically into the tumor cells, and control its expression in the tumor cells using such as promoter sensitivity [31]. In this paper, we use a diphtheria toxin A (DTA) as a target therapeutic gene, an immunotoxin that is widely used in gene therapy due to its role in protein synthesis inhibition [32]. The DTA could inactivate elongation factor 2 by adenosine diphosphate ribosylation, as well as inhibit protein translation in the cells and trigger apoptosis [33]. This gene has been used to target various cancers, including pancreatic cancer [34,35], ovarian cancer [36], glioblastoma [37], and bladder cancer [38,39], in combination with various delivery methods, including an integrase-deficient lentiviral vector [32] and plasmid DNA [35,37,38]. Promising effects on tumor cell growth have been reported, but, because of its strong inhibitory effect on the protein synthesis, its applications for cancer therapy are based on tumor cell selectivity with specific promoters [39,40,41,42] and fused protein [43,44]. For HCC, a limited number of studies have examined HCC cell lines [37,42], wherein cell line-transplanted mice have been used to test its efficacy in vivo. Therefore, this study examines the antitumor effect of DTA-expressing plasmid in HCC in vitro and in vivo with alpha-fetoprotein (AFP) promoters for HCC cell-specific gene expression. The plasmid is delivered by hydrodynamic gene delivery [45,46,47,48,49] to the liver, carrying the yes-associated protein (YAP) gene expression induced HCC. Our results demonstrate that the overexpression of AFP promoter-controlled DTA inhibited cell growth through the inhibition of protein synthesis in an AFP-dependent manner, significantly decreased the occurrence of YAP-induced HCC in normal mice liver; moreover, it inhibited the tumor marker increase, suggesting the clinical applicability of the procedure for HCC gene therapy.

## 2. Results

### 2.1. Development of DTA-Expressing Plasmid

**Figure 1:**
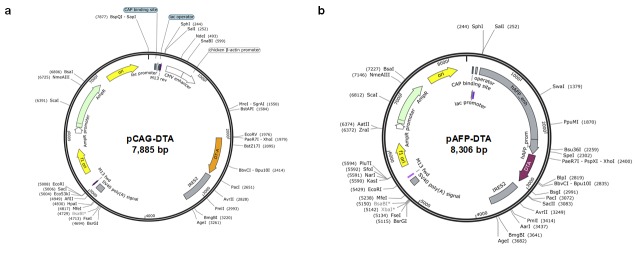
Development of diphtheria toxin fragment A (DTA)-expressing plasmid. The DTA expression vector, containing (**a**) a chicken β-actin promoter, cytomegalovirus enhancer, and an internal ribosome entry site (IRES) (pCAG–DTA), and (**b**) a human alpha-fetoprotein (AFP) promoter, cytomegalovirus enhancer, and an IRES (pAFP–DTA).

The complementary DNA of DTA was inserted into the pIRES2 plasmid vector containing an internal ribosome entry site (IRES). The expression of DTA was under the control of a chicken β-actin promoter and cytomegalovirus enhancer. The plasmid was named pCAG–DTA; it had a size of 7885 bp (Figure 1a). Another plasmid was developed wherein complementary DNA of DTA was inserted into the pIRES2 plasmid, in which the expression of DTA was controlled under the human AFP promoter. This plasmid was named pAFP–DTA; it had a size of 8306 bp (Figure 1b).

### 2.2. Effect of DTA Gene Expression in Mice Liver on Protein Synthesis

**Figure 2:**
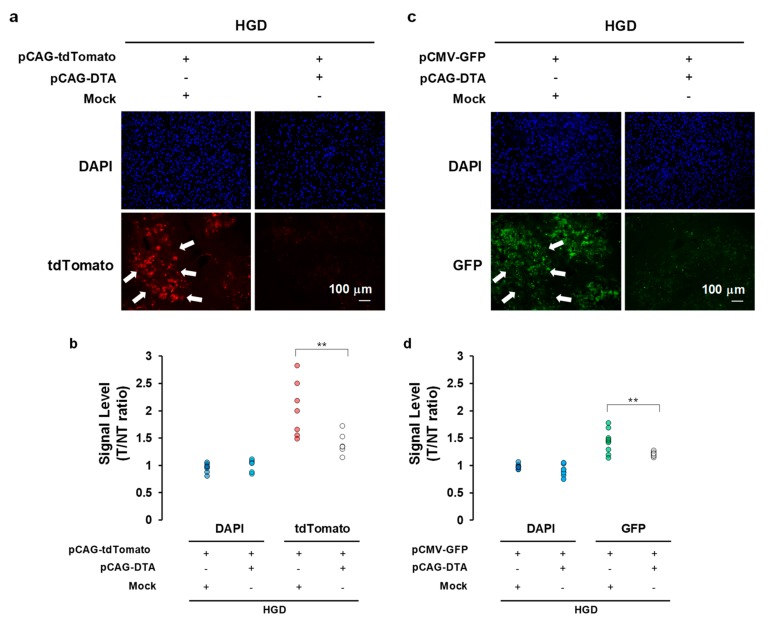
Effect of DTA on protein synthesis inhibition. (**a**) Effect of DTA on tdTomato expression in mice hepatocytes 12 h after the hydrodynamic gene delivery of pCAG–tdTomato with pCAG–DTA. (**b**) A quantitative analysis of tdTomato signal level determined with the ratio of the signal (T/NT ratio) in the transfected (T) liver and non-transfected (NT) liver collected from the mock transfected liver. (**c**) Effect of DTA on green fluorescent protein (GFP) expression in mice hepatocytes 12 h after the hydrodynamic gene delivery of pCAG–tdTomato with pCAG–DTA. (**d**) A quantitative analysis of the GFP signal level determined with the T/NT ratio. White arrows indicate expression of tdTomato and GFP. Scale bar represents 100 µm. The values from the two sections of three mice are shown. ** *p* < 0.01. Student’s *t*-test. Mock, empty vector; DTA, diphtheria toxin A.

To examine the effect of DTA on the protein synthesis inhibition, the amount of transgene-derived fluorescent protein was assessed with the expression of the DTA protein in vivo. Either the tdTomato-expressing plasmid (pCAG–tdTomato) or the green fluorescent protein (GFP)-expressing plasmid (pCMV–GFP) was hydrodynamically delivered to the mice liver with or without the DTA-expressing plasmid (pCAG–DTA) (Figure 2). Although the DAPI stain showed no difference 12 h after the hydrodynamic gene delivery, significant inhibition was evident of the tdTomato signal ratio to the control (non-transfected) liver from 2.28 ± 0.19 to 1.39 ± 0.08 (*p* < 0.05) (Figure 2a,b). A similar result was obtained with the GFP level from 1.44 ± 0.04 to 1.21 ± 0.01 (*p* < 0.05) (Figure 2c,d). These results indicate the inhibitory effect of overexpression of the DTA gene by a hydrodynamic procedure on protein synthesis in the mice liver cells. The liver injected with pCAG-DTA showed increase in terminal deoxynucleotidyl transferase (TdT)-mediated dUTP nick end labeling staining 12 h after the injection; this indicated that the inhibition of protein synthesis by DTA, with no selection, caused apoptotic changes in the transfected cells (Figure S1).

### 2.3. Effect of DTA Expression in HCC Cells on the Cell Growth

**Figure 3:**
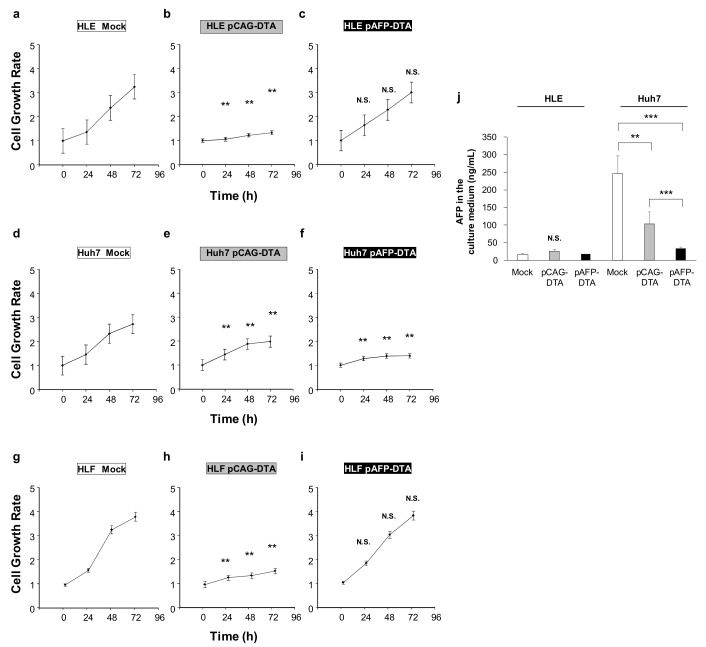
Effect of DTA on hepatocellular carcinoma (HCC) cell growth. The cell growth of HCC cell lines and permanent clones overexpressing DTA determined by MTT assay. (**a**–**c**) Cell growth of HLE and (**d**–**f**) Huh7, and (**g**–**i**) HLF cell lines transfected with mock or CAG–DTA or pAFP–DTA. The values represent mean ± standard deviation (five samples from each group of three, where *n* = 15 for each group at different time points). ** *p* < 0.01 and no statistical significance (N.S.). Two-way ANOVA followed by Bonferroni’s multiple comparison test. (**j**) A concentration of AFP in the cell culture medium at 72 h after transfection was quantified by ELISA. The values represent mean ± standard deviation (*n* = 3 for each group). ** *p* < 0.01, *** *p* < 0.001, and N.S. One-way ANOVA followed by Bonferroni’s multiple comparison test.

To examine the effect of DTA on the cell growth of liver cancer cells, we transfected DTA-expressing plasmids into the HCC cell lines of HLE, Huh7, and HLF, and assessed the cell growth (Figure 3a–i). In comparison with the HLE and Huh7 cells transfected with the plasmid lacking the DTA gene (Figure 3a,d), the cells transfected with the pCAG–DTA plasmid showed significant inhibition of growth rate under the normal culture condition determined by the MTT assay (Figure 3b,e). In comparison, the MTT assay showed no significant inhibition of cell growth in the HLE transfected with pAFP–DTA (Figure 3c), whereas the growth of Huh7 cell line, which is the cell line responsible for producing AFP, was significantly inhibited (Figure 3f). The HLF showed a similar pattern to that of HLE (Figure 3g–i). The concentration of AFP in the cell culture medium showed significantly high levels in the mock transfected Huh7 cell lines, and was significantly inhibited by pAFP–DTA transfection (*p* < 0.001). Although the pCAG–DTA transfection also inhibited the increase of AFP in the medium, the effect of inhibition was more significant in transfecting pAFP–DTA (*p* < 0.001), suggesting the efficacy of AFP promoter selectivity with respect to expressing DTA (Figure 3j). Increase in AFP was not seen in the HLE cultured media. These results indicated that the DTA expression is effective in inhibiting HCC cell growth and can be controlled by AFP promoter selectivity (Figure 3).

### 2.4. Effect of DTA on Tumor Growth In Vivo

**Figure 4:**
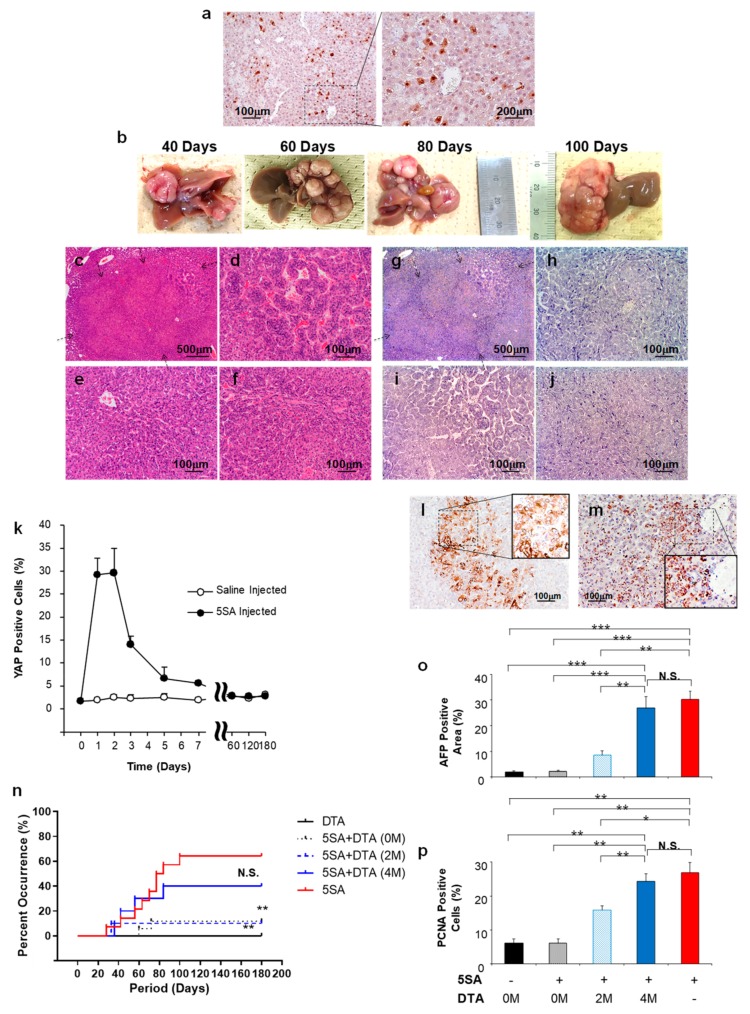
Effect of DTA on HCC mice model. (**a**) Expression of yes-associated protein (YAP) in the liver 3 days after the hydrodynamic gene delivery of YAP-expressing plasmid (5SA). (**b**) Representative images of time-dependent liver–tumor occurrence and sizes. (**c**–**f**) Hematoxylin and eosin staining and (**g**–**j**) immunohistochemical staining of YAP of the liver tumor. (**k**) Time dependent changes in YAP expression in the liver after 5SA injection. Quantification was performed by measuring the integrated density in pixels using the ImageJ software (version 1.6.0_20, National Institutes of Health, Bethesda, MD, USA). The values represent mean ± standard deviation (*n* = 5 for each group). (**l**,**m**) Immunohistochemical staining of the AFP of the liver–tumor developed 40 (**l**) and 100 days (**m**) after the delivery of 5SA. (**n**) Cumulative HCC occurrence curve in the liver of 5SA-injected mice generated by the Kaplan–Meier method. The occurrence rate of HCC was compared with 5SA injected with no treatment (5SA, red solid line), treated with pAFP–DTA immediately after treatment (5SA + DTA (0 M), black dot line), 2 months after treatment (5SA + DTA (2 M), blue dot line), 4 months after treatment (5SA + DTA (4 M), blue solid line), and control (pAFP–DTA with no ¬YAP induction, DTA, black solid line). *n* = 15 for each group. ** *p* < 0.01 and N.S. compared with the 5SA-delivered mice with no treatment group (red solid line). Log-rank test. Quantitative analysis of area with positive staining for AFP (**o**) and proliferating cell nuclear antigen (PCNA) (**p**) in the tumors of each group developed 180 days after YAP-expressing plasmid (5SA) delivery with/without gene therapy of pAFP–DTA. Quantification was performed measuring the integrated density in pixels using the ImageJ software (version 1.6.0_20, National Institutes of Health). The values represent mean ± standard deviation (*n* = 5 for each group; same number of liver tissue specimens were assessed for no 5SA-delivered group). * *p* < 0.05; ** *p* < 0.01; *** *p* < 0.001; and N.S. One-way ANOVA followed by Bonferroni’s multiple comparison test. 0 M, 2 M, and 4 M, at 0, 2, and 4 months after the delivery of pAFP–DTA.

To examine the effect of DTA on tumor growth in vivo mice models, an HCC model mouse was developed by transferring the YAP-expressing plasmid (5SA) by hydrodynamic gene delivery. The successful expression of YAP protein was confirmed 3 days after the hydrodynamic injection (Figure 4a), with a time-dependent increase in occurrence and liver–tumor size (Figure 4b). Moreover, approximately 6 months after the delivery of 5SA, 70% of the mice showed liver–tumor occurrence, which was histologically diagnosed as HCC (Figure 4c) with various histological differentiation (Figure 4d–f). However, the tumor developed 6 months after the delivery showed no significant expression of YAP (Figure 4g–j). Time dependent changes in YAP expression in the liver tissue (Figure 4k) showed significant increase to 30% of cells, 2–3 days after hydrodynamic injection; the levels decreased to the background level within a week. These findings suggest that the initiation of YAP expression is essential in the proposed mice HCC model. In addition, the AFP protein expression was confirmed in the tumor cells developed at various time points (40 days after 5SA delivery in Figure 4l and 100 days after in Figure 4m). The time-dependent tumor occurrence is summarized in Figure 4m when no treatment was administered (red solid line); for these mice models, DTA-expressing plasmid under the control of AFP promoter (pAFP–DTA) was transferred to the liver at 0, 2, and 4 months after 5SA delivery by the hydrodynamic gene delivery procedure, and the control group was hydrodynamically injected with pAFP–DTA, and examined for 6 months (Figure 4n, black solid line). All groups were assessed according to the tumor occurrence rates. Although significant inhibition of tumor development by DTA gene delivery was evident when administered at 0 (Figure 4n, black dotted line) and 2 months (Figure 4n, blue dotted line) after 5SA delivery (*p* < 0.05), no such effect was evident 4 months after 5SA delivery (Figure 4n, blue solid line). To assess the tumor types and morphology, the expression of AFP (Figure 4o) and proliferating cell nuclear antigen (PCNA) (Figure 4p) were examined. The time dependent increase in these markers was confirmed after 5SA injection; in addition, the AFP expression and cell proliferation were inhibited by pAFP–DTA delivery. These results demonstrate the antitumor effect of DTA in the YAP-induced mice HCC model, especially in the early stages of tumor development.

### 2.5. Effect of DTA on HCC Tumor Marker In Vivo

**Figure 5:**
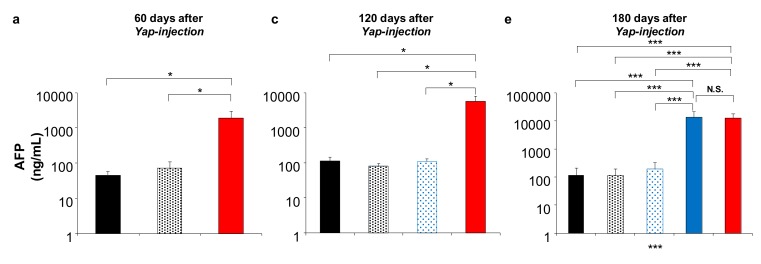

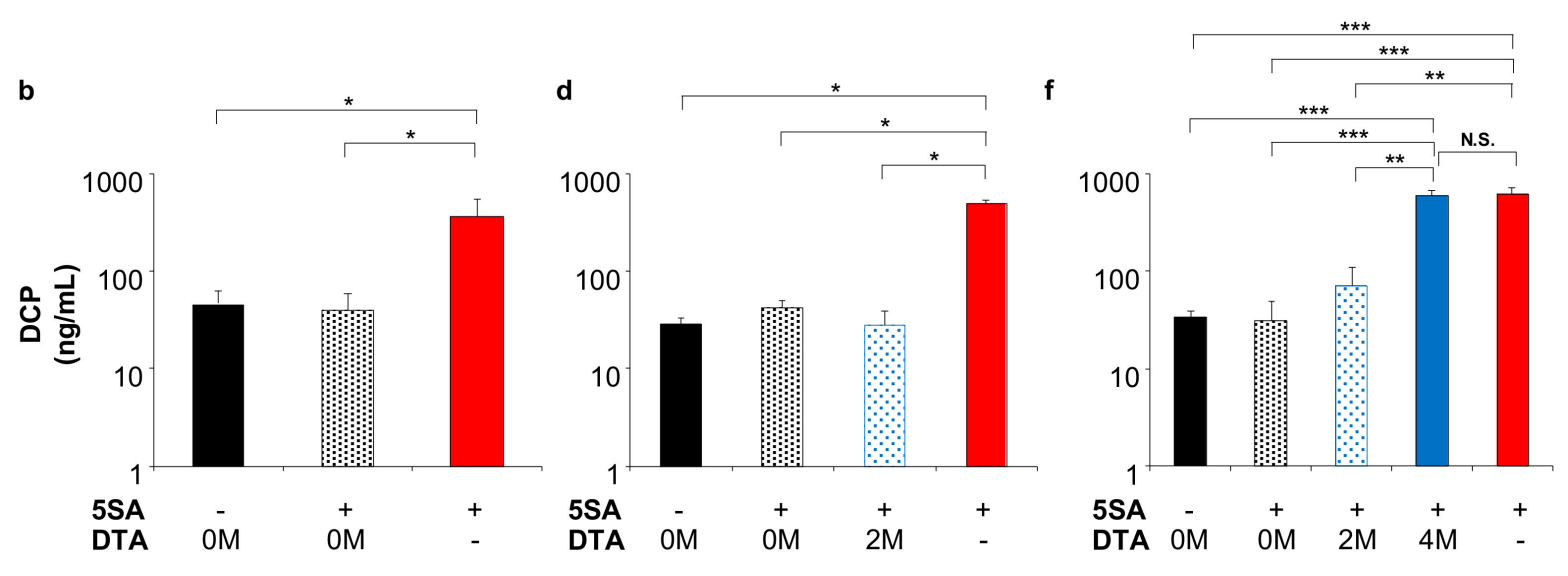
Tumor markers in YAP-induced HCC mice model treated with pAFP–DTA. Serum concentration of AFP and des-gamma carboxyprothrombin (DCP) quantified at 60 days (**a**,**b**), 120 days (**c**,**d**), and 180 days (**e**,**f**) after YAP-expressing plasmid (5SA) delivery with/without the gene therapy of pAFP–DTA by ELISA. The values represent mean ± standard deviation (*n* = 5 for each group). * *p* < 0.05; ** *p* < 0.01; *** *p* < 0.001; and N.S. One-way ANOVA followed by Bonferroni’s multiple comparison test. 0 M, 2 M, and 4 M, at 0, 2, and 4 months after the delivery of pAFP–DTA.

To confirm the effect of DTA on tumor growth inhibition, a serum tumor marker of AFP and des-gamma carboxyprothrombin (DCP) was examined in the mice models at 60, 120, and 180 days after the delivery of the YAP gene, with and without the gene therapy of pAFP–DTA at appropriate time points (Figure 5). In the HCC model, AFP, and DCP increase to 1899.1 ± 1074.4 and 374.5 ± 1074.4 ng/mL, respectively, 60 days after the delivery of 5SA (red solid bars, Figure 5a,b); at 120 days, they continue to increase to 5860.7 ± 2293.7 and 549.7 ± 103.1 ng/mL, respectively (red solid bars, Figure 5c,d); at 180 days, they continue to increase to 13,448.2 ± 8787.2 and 590.6 ± 306.7 ng/mL, respectively (red solid bars, Figure 5e,f). Normal mice treated with pAFP–DTA showed sustained low levels of AFP and DCP (~100 and 50 ng/mL, respectively) over the entire study period (black solid bars in Figure 5). At 60, 120, and 180 days, the HCC mice model treated with pAFP–DTA after 5SA delivery showed AFP levels of 71.4 ± 37.1, 82.5 ± 14.5, and 117.7 ± 92.0 ng/mL, as well as DCP levels of 40.1 ± 18.5, 41.7 ± 7.5, and 31.2 ± 17.4 ng/mL, respectively (black dotted bars in Figure 5). Indeed, these values are significantly lower than those of the non-treated mice (red solid bars in Figure 5). Moreover, at 120 and 180 days, the HCC mice model treated with pAFP–DTA 2 months after 5SA delivery showed AFP levels of 110.4 ± 18.5 and 193.5 ± 129.5 ng/mL and DCP levels of 27.3 ± 11.3 and 70.4 ± 37.7 ng/mL, respectively (blue dotted bars in Figure 5). Again, these values are significantly lower than those of the non-treated mice (red solid bars in Figure 5). In addition, at 180 days, the HCC mice model treated with pAFP–DTA 4 months after 5SA delivery showed an AFP level of 13,624.4 ± 7984.4 ng/mL and a DCP level of 587.2 ± 93.0 ng/mL, which is similar to that of the non-treated mice (blue solid bars in Figure 5). These results suggest that DTA gene delivery to the liver, in combination with HCC, inhibited both tumor occurrence and tumor growth.

### 2.6. Safety of the DTA Gene Therapy with Promoter Selectivity

**Figure 6:**
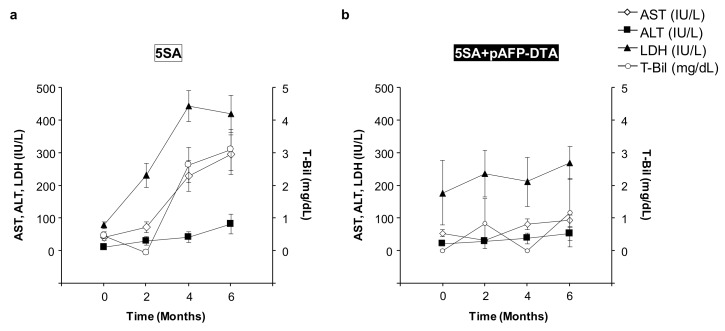
Time-dependent course of serum biochemical factors. Serum concentrations of aspartate transaminase (AST), alanine aminotransferase (ALT), lactate dehydrogenase (LDH), and total bilirubin (T-Bil) in (**a**) mice delivered with YAP-expressing plasmid (5SA) and (**b**) mice treated with pAFP–DTA just after the delivery of 5SA. The values represent mean ± standard deviation (*n* = 3 for each group).

Changes of serum biochemical factors were assessed to determine the safety of delivering the AFP promoter-controlled DTA gene into the mice liver (Figure 6). Although the serum levels of aspartate transaminase (AST), alanine aminotransferase (ALT), lactate dehydrogenase (LDH), and total bilirubin (T-Bil) increased after 5SA delivery, which was probably due to the cytotoxicity and tumorigenicity of the liver (Figure 6a), they did not significantly increase in the mice treated with pAFP–DTA after 5SA delivery over the entire 180-day study period (Figure 6b). These results suggest that gene therapy using the DTA gene under the control of an AFP promoter to the liver carrying high levels of oncogene is safe.

## 3. Discussion

Conventional therapeutic options for HCC, such as liver transplantation, ablation, chemoembolization, systemic chemotherapy, and molecularly targeted agents, can be effective [2]; however, due to the heterogeneity of tumor cells [12,13,14,15], patients often become resistant to said treatments, which results in high recurrence rates, especially in advanced stages. Moreover, HCC is the fourth most common cause of cancer-related deaths worldwide [2], and, accordingly, the establishment of a novel therapeutic strategy is essential. With the expansion of genetic information obtained by the NGS and the development of molecular analyses, basic research focusing on strategies that target tumor-related genes, proteins inducing tumor cell death are significantly increased. Among these novel focuses, cancer gene therapy for HCC is increasing in popularity [26]. This strategy includes the in vivo modification of tumor suppressor and oncogenes, induction of suicide genes into the tumor cells, and ex vivo gene transfer of T-cells, which attack the tumor cells targeting the proteins expressed on the tumor cell surface [17,24,25,27].

DT is a toxin produced by *Corynebacterium diphtheria* [50], consisting of 535 amino acids of 62 kDa Y-shaped molecules [50]. This protein consists of two fragments, of which the fragment A (DTA) in the N-terminus includes a catalytic domain that stops the protein synthesis in the cells and induces a cytolethal effect [51]. The fragment B in the C-terminus contains the transmembrane domain and the receptor-binding domain, which contribute to transferring toxin into the susceptible cells [51]. The catalytic domain in DTA binds to nicotinamide dinucleotide in the cytoplasm of the DTA transferred/expressed cells, and then transfers an adenosine diphosphate ribosyl moiety to the elongation factor in the cells, and inhibits the protein synthesis [33,51]. On the basis of these cytolethal effects, DTA is useful in cancer gene therapy. Several clinical trials have been conducted for malignant lymphoma, leukemia, glioblastoma, breast cancer, and lung cancer; moreover, in vivo and in vitro research have also been conducted for brain tumors, leukemia, prostate cancers, breast cancer, cervical cancer, metastatic cancer, ovarian cancer, and colon cancer targeting IL-2, GM-CSF, IL-3, EGF, CD19, CD22, IL-13, and IL-7 [33]. For HCC, only two in vitro [52,53] and two xenograft mice model studies [37,42] have been reported; clinical trials have not been conducted or approved to date [33]. The major problem in utilizing DTA for cancer gene therapy is associated with damage caused to the tumor-surrounding tissue: when DTA gene is delivered to normal cells, a severe toxic effect may follow. Indeed, this was observed in our study (Figure 1 and Figure 2). To address this issue, the application of the tumor-specific promoters was utilized to express DTA protein specifically in the tumor cells. For HCC, insulin-like growth factor promoters [37] and an AFP promoter [42] were used for the xenograft models, and, for in vitro studies, a human telomerase reverse transcriptase promoter and a synthetic β-catenin-dependent promoter were used. Although previous studies have shown the potential safety and efficacy of using DTA for HCC gene therapy, reports are currently lacking with respect to the efficacy of DTA gene therapy in in vivo mice HCC models. Therefore, we conducted the in vivo study examining the DTA gene therapy for the HCC mice model induced by the ¬YAP gene overexpression. The results suggest that the effective AFP promoter controlled the cytolethal effect in HCC cell lines, showing the protein synthesis inhibition and growth inhibition in a promoter-controlling manner (Figure 1 and Figure 2). In addition, the YAP-induced HCC mice model showed significant tumor occurrence inhibition, suppressing the tumor markers of AFP and DCP when DTA was administered within the first 4 months after the initiation of oncogene (Figure 3 and Figure 4).

For the tumor-specific gene delivery, various strategies use viral and nonviral gene delivery procedures, including intratumoral injection, intra-arterial injection, intravenous injection, intraportal injection, and intramuscular injection [26,54,55]. Among these, we used the hydrodynamics-based gene delivery procedure [48,49] for HCC gene therapy, which was tested in rat liver fibrosis models [56,57]. The major advantages of this procedure are simplicity, reproducibility, and ease of gene preparation with respect to being delivered. The genes can be delivered to 30–40% of cells, mostly the hepatocytes in the liver [54]. As a result, the YAP gene-delivered liver showed an HCC occurrence of 70–80% [58,59], and the tumor showed histologically heterogeneous tumors similar to human HCC within a short period of 6 months. YAP itself has also been extensively studied [60,61,62,63,64,65,66,67]. It was shown to contribute to the activation of various gene expressions [60] and to the determination of cell fate [60]. It was also shown to contribute to the metastasis of malignant cells [61,67], development of malignant liver tumors [62,63,64,65], and liver regeneration [66]. In addition, the Yap knockout mice are embryonic lethal, while liver conditional knockout mice showed delayed liver regeneration after partial hepatectomy, revealing the importance of Yap protein in maintaining biological homeostasis [66]. On the other hand, continuous overexpression of Yap in transgenic mice resulted in hepatic carcinogenesis characterized by heterogeneous HCC-like, intrahepatic cholangiocellular carcinoma (ICC)-like, and mixed type-like liver cancers [62,63,64]. Therefore, the Yap–HCC mice model has been used to examine the YAP as a target of gene therapy [59], and a recent report clearly demonstrated that the Yap protein itself is a promising therapeutic target in the context of liver cancer.

In our study, we utilized a hydrodynamic tail vein injection to deliver naked plasmid DNA to the liver of mice; therefore, YAP gene expression was transient and showed a significant decrease within a week and returned to background level (Figure 4k). These findings are consistent with those of a previous study [60]. Therefore, hepatic carcinogenesis in our study was not only dependent on Yap expression at the initial stage of carcinogenesis, but also on the activation of other oncogenic pathways, including Ras, AKT, and c-Myc, as previously reported [62,64]. This resulted in heterogeneous tumors (Figure 4b–j). Therefore, we selected DTA as a therapeutic gene for gene therapy of these heterogeneous tumors to inhibit protein synthesis, which has been used in various cancers [32,33,34,35,36,37,38,39,40,41,42,43,44], and used an AFP promoter to control its expression within the AFP-expressing HCC cells.

To apply this method to the human liver, modified procedure has been tested in the large animals, including pigs [45,46], dogs [47], and baboons (manuscript in preparation). The development of appropriate toxin-based constructs can treat heterogeneous cancers, which, currently, cannot be cured using conventional therapeutic options.

This study was characterized by some limitations. Although we used YAP*-*induced HCC models, other models can be used to examine the efficacy and safety of the toxin-based gene therapy. Moreover, the liver tissues delivered with the YAP gene were sectioned to identify small tumors under a microscope; however, bioluminescence imaging should be used to detect tumors in vivo. In addition, the promoter used in this study was limited to an AFP. We need to consider that YAP is a potential therapeutic target and a useful prognostic marker in the context of HCC. In addition, the clinical feasibility of the procedure can be tested in more heterogeneous liver tumors, such as ICC-like and mixed type-like tumors, developed in the Yap transgenic mice liver. To address these limitations, further studies are required to examine the efficacy of various DTA-expressing constructs with various promoters in combination with imaging modalities for various HCC mice models.

In conclusion, our results demonstrate that DTA-expressing plasmid inhibited proliferation of HCC cells and DTA expression in an AFP-promoter-dependent manner and reduced the growth of HCC in vivo and decreased the tumor markers of AFP and DCP. Along with the analyses of serum biochemistry, our results demonstrated the safety of hydrodynamics-based gene delivery of DTA-expressing plasmid. Our findings suggest the clinical applicability of the procedure for HCC gene therapy.

## 4. Materials and Methods

### 4.1. Animals

Animal experiments were approved by, and conducted in full compliance with, the regulations of the Institutional Animal Care and Use Committee at Niigata University, Niigata, Japan. Male C57BL/6J mice (*n* = 100, 8 weeks old, and 25–30 g) were purchased from CLEA Japan, Inc. (Tokyo, Japan). Mice were housed under standard conditions at a temperature of 20–23 °C, humidity of 45–55%, and in specific pathogen-free facilities. Mice were given hydrodynamic injections as previously described [48,49]. In summary, under anesthesia by isoflurane, an injection needle (27 gauge, Terumo, Shibuya-ku, Tokyo, Japan) was inserted to the tail vein, and 10% body weight volume of normal saline containing plasmid DNA (10 μg/mL) was hydrodynamically injected with a flow rate of 1 mL/s.

### 4.2. Plasmids

The YAP-expressing plasmid (5SA) was constructed using the full-length complementary DNA of human YAP ligated into *Xba*I restriction sites of the expression vector of pLIVE vector (Mirus Bio., Madison, WI, USA). The DTA expression vector, which contained a chicken β-actin promoter, cytomegalovirus enhancer, and an IRES (pCAG–DTA) as well as a human AFP promoter, cytomegalovirus enhancer, and an IRES (pAFP–DTA), was generated through a multi-step and ligation-based cloning procedure using the full-length complementary DNA of DTA. pCAG–tdTomato and pCMV–GFP were generated, cloning the complementary DNA of tdTomato and GFP into the plasmids containing a chicken β-actin promoter and cytomegalovirus enhancer and an IRES and cytomegalovirus promoter. A circular plasmid map was generated using the SnapGene Viewer. The plasmid was purified using the Plasmid Mega Kit (Qiagen, Hilde, Germany). The purity of the plasmid preparation was checked by absorbency at 260 and 280 nm and 1% agarose gel electrophoresis.

### 4.3. Cells

Human hepatoma HLE, HLF, and Huh7 cell lines were purchased from the Japanese Collection of Research Bioresources Cell Bank (National Institutes of Biomedical Innovation, Health and Nutrition, Ibaraki, Osaka, Japan) and were cultured in minimum essential medium, which contained 10% fetal bovine serum and 100 IU/mL of penicillin and streptomycin. Cells were incubated in a 5% CO_2_ humidified incubator at 37 °C. These cells were transfected with either mock or DTA-expressing vectors using FuGENE HD transfection reagent (Promega, Madison, WI, USA) according to the instructions supplied.

### 4.4. Cell Growth Assay

Cells were plated in 96-well tissue culture dishes (2 × 10^4^ cells per well) in 100 µL of the aforementioned medium. The 3-(4,5-dimethylthiazol-2-yl)-2,5-diphenyltetrazolium bromide (MTT) reagents were added to the cells at the indicated times after the treatment; the cells were then counted by means of the Premix WST-1 Cell Proliferation Assay System (Takara Inc., Kyoto, Japan), according to the instructions supplied.

### 4.5. Fluorescence Image

Frozen liver embedded in Optimal Cutting Temperature compound (Sakura Finetek, Torrance, CA, USA) was used to assess the fluorescent signals of the tdTomato and GFP proteins using an excitation filter 543 nm wide and 489 nm long. Nuclei were counterstained with 4′,6-diamidino-2-phenylindole (DAPI). The fluorescent images were obtained with a Zeiss Axiovert 200M microscope (Carl-Zeiss, Oberkochen, Germany). Quantitative analysis was conducted with respect to the fluorescent signal level, which was determined according to the ratio of the signal in the transfected liver and the non-transfected liver collected from the mock transfected liver.

### 4.6. Histological Analysis

Tissue samples for hematoxylin, eosin, and immunohistochemical staining were collected from each group at appropriate time points after the procedures. Livers were sectioned carefully with a thickness of 1 mm after a week of fixation in 10% formalin to identify any tumors; then they were embedded in paraffin. Immunohistochemical staining for YAP, AFP, and PCNA was conducted with an anti-YAP antibody (No. 4912; Cell Signaling Technology, Danvers, MA, USA) at 1:100 dilution and an anti-AFP antibody (ab46799; Abcam, Cambridge, UK) at 1:100 using the Vectastain Elite ABC rabbit IgG kit (PK-6101, Vector Laboratories, Burlingame, CA, USA) and anti-PCNA (No. 2586; Cell Signaling Technology, Danvers, MA, USA) at 1:10,000 dilution using the Vectastain Elite ABC mouse IgG kit (PK-6102, Vector Laboratories, Burlingame, CA, USA) and 3,3′-diaminobenzidine chromogen tablets (Muto Pure Chemicals, Tokyo, Japan). The determination and diagnosis of the liver tumor was conducted by three hepatologists and/or pathologists.

### 4.7. Serum Biochemical Analysis

Biochemical analysis of serum levels of aspartate transaminase (AST), alanine aminotransferase (ALT), lactate dehydrogenase (LDH), and total bilirubin (T-Bil) were conducted in Oriental Yeast Co. Ltd. (Siga, Japan) serum. AFP and des-gamma carboxyprothrombin (DCP) levels were determined by enzyme-linked immunosorbent assay (ELISA) using human MMP13 ELISA kit (ELH-MMP13, RayBiothech Inc., Norcross, GA, USA), a mouse AFP Quantikine ELISA kit (DAFP00, R&D Systems, Inc., Minneapolis, MN, USA), and a mouse abnormal prothrombin/DCP ELISA kit (MBS268938, MyBioSource, Inc., San Diego, CA, USA).

### 4.8. Statistical Analysis

The obtained data were analyzed using either Student’s *t*-test or the one-way and two-way factor repeated-measure analysis of variance (ANOVA), followed by Bonferroni’s multiple comparison test. The cumulative liver–tumor occurrence curve was generated by the Kaplan–Meier method; the occurrence rates were compared using a log-rank test. GraphPad PRISM 7 (GraphPad Software, La Jolla, CA, USA) was used for said analyses, and a *p* value ≤ 0.05 was considered to indicate statistical significance.

## 5. Conclusions

In conclusion, our study demonstrated that DTA is safe and effective with respect to inhibiting HCC growth under the control of an AFP promoter. This was the first report to document the effectiveness of toxin-based suicide cancer gene therapy for HCC in in vivo mice models.

## Supplementary Materials

The following are available online at https://www.mdpi.com/2072-6694/12/2/472/s1, Figure S1: TUNEL staining of the mice liver hydrodynamically injected with 10% body weight solution of saline (a) or pCAG-DTA.
